# Prevalence and impact of autoimmune thyroid disease on myasthenia gravis course

**DOI:** 10.1002/brb3.537

**Published:** 2016-08-04

**Authors:** Justyna Kubiszewska, Beata Szyluk, Piotr Szczudlik, Zbigniew Bartoszewicz, Małgorzata Dutkiewicz, Maksymilian Bielecki, Tomasz Bednarczuk, Anna Kostera‐Pruszczyk

**Affiliations:** ^1^ Department of NeurologyMedical University of WarsawWarsawPoland; ^2^ Department of Internal Medicine and EndocrinologyMedical University of WarsawWarsawPoland; ^3^ Department of Immunology, Biochemistry and NutritionMedical University of WarsawWarsawPoland; ^4^ Department of PsychologySWPS University of Social Sciences and HumanitiesWarsawPoland

**Keywords:** autoimmune thyroid diseases, Graves’ disease, Hashimoto's thyroiditis, myasthenia gravis

## Abstract

**Objectives:**

Autoimmune thyroid diseases (ATDs) frequently accompany myasthenia gravis (MG) and may influence its course. We aimed to determine the association and impact of ATD with early‐ (<50 years), late‐onset MG, or thymoma‐MG.

**Materials and Methods:**

Prevalence of ATD was measured in a cross‐sectional study of 343 consecutive patients with MG (236 F, 107 M) aged 4–89 years; 83.8% were seropositive, in 2.9%, anti‐MuSK antibodies were detected. Concentrations of antithyroid peroxidase antibodies, antithyroglobulin antibodies, antithyrotropin receptor antibodies, and TSH level were measured in all patients. MG clinical course, treatment received, and treatment results were evaluated.

**Results:**

Autoimmune thyroid diseases were diagnosed in 92 (26.8%) of MG patients including 4.4% with Graves (GD), 9% with Hashimoto thyroiditis (HT), and 13.4% with antithyroid antibodies only. GD patients had ocular symptoms more often than patients with antithyroid antibodies or HT (*p* = .008). ATD prevalence was comparable in MG with early and late onset, while non‐ATDs were more frequent in thymoma‐MG (*p* = .049). Immunosuppressive therapy was less frequently needed in the patients with MG and ATD, indirectly indicating milder MG course (*p* = .005). Risk of myasthenic crisis and the results of treatment did not differ between patients with and without ATD.

**Conclusions:**

Autoimmune thyroid diseases are frequently accompanied by early‐and late‐onset MG, while thymoma‐MG is related to higher risk of non‐ATD. Myasthenia coexisting with ATD follows milder course than MG alone.

## 1 Introduction

Myasthenia gravis (MG) is a rare antibody‐mediated autoimmune disease of the neuromuscular junction, with incidence 0.25–2.00 per 100,000 per year (Verschuuren, Palace, & Erik Gilhus, 2010; Vincent, Palace, & Hilton‐Jones, 2001).

In MG, distinct subgroups were defined: early‐ or late‐onset MG (EOMG or LOMG; onset before or after age 50 years, respectively) or MG associated with thymoma (T‐MG). Autoimmune thyroid diseases (ATDs) are a heterogeneous group of disorders; the three major phenotypes are: (1) patients with hyperthyroidism due to Graves’ disease (GD); (2) patients with hypothyroidism due to Hashimoto's thyroiditis (HT); and (3) euthyroid patients with positive antithyroid autoantibodies (Ban et al., 2008; Duntas, 2008; Kiessling, Pflughaupt, Ricker, Haubitz, & Mertens, 1981). Prevalence of antithyroid autoimmunity accompanying MG varies in reported series depending on the methods used to identify ATD in the studied cohort from 10% to 20% of MG patients (Christensen et al., 1995; Nakata et al., 2013; Thorlacius, Aarli, Riise, Matre, & Johnsen, 1989).

Hormonal disequilibrium (both hyper‐ and hypothyroidism) as a cause of MG exacerbation was a subject of numerous case reports (Konno et al., 2009; Sarkhy, Persad, & Tarnopolsky, 2008). There is little data on the impact of ATD on the long‐term course of MG.

The aim of our study was to determine the prevalence and impact of ATD on EOMG, LOMG, or thymoma‐associated MG in a cohort of Polish patients.

## 2 Materials and Methods

### 2.1 Studied groups

Cross‐sectional study of 343 consecutive patients (236 women and 107 men) with MG was performed. The patients were enrolled to the study after informed consent from 1st May 2009 till 31st December 2012 at a single neuromuscular center. Study was approved by the local Ethics Committee. Mean age at MG onset was 39 years (*SD:* 22.4, range: 2–89) and mean age at enrollment was 47.6 (*SD:* 20.4, range: 4.5–89.1). MG diagnosis was based on the history, clinical presentation, results of repetitive nerve stimulation test or SFEMG, or serological tests: acetylcholine receptor antibody (ARAb), MuSKAb (RIA, DLD Diagnostica Germany). Data of the patients were collected in the form of questionnaire. The course of the disease including the first symptoms, clinical severity graded with MGFA scale (Jaretzki et al., 2000a), and presence of ocular symptoms was noted in all patients. Result of thymus pathology, the treatments applied, and the state of intervention at last visit were determined. The severity of clinical symptoms was graded with MG Foundation of America (MGFA) scale (Jaretzki et al., 2000a, 2000b) at the beginning of the disease, the maximum, and at the last visit.

The diagnosis of GD was confirmed by the presence of: (1) hyperthyroidism, (2) detectable TSH receptor autoantibodies (TRAB), (3) and/or increased radioiodine uptake, (4) and/or the presence of clinically evident orbitopathy (Kurylowicz et al., 2007; Ploski et al., 2010). None of the GD patients in the study received GCS therapy for Graves’ orbitopathy.

The diagnosis of HT was confirmed by the presence of: (1) hypothyroidism; (2) detectable antithyroid peroxidase antibodies (TPO) antibodies and/or antithyroglobulin antibodies (TG); (3) and/or decreased echogenicity of the thyroid on US examination.

Thyroid antibodies group was defined by the presence of: (1) euthyroidism; (2) detectable anti‐TPO and/or anti‐TG antibodies; (3) and/or decreased echogenicity of the thyroid in US examination. Nonautoimmune thyroid disease patients had history of hypo‐ or hyperthyroidism or toxic nodular goiter.

### 2.2 Measurement of thyroid autoantibodies and TSH

In all patients, concentrations of anti‐TPO, antithyroglobulin antibodies (TG), anti‐TSHR (thyrotropin receptor antibodies), and TSH level were measured. TSH level, anti‐TPO, anti‐TG, and anti‐TSHR in the serum samples were determined based on the high‐sensitivity technology of the electrochemiluminescence using streptavidin‐coated paramagnetic microparticles using automated immunodiagnostic analyzer Elecsys 2010, Roche Diagnostics. The values for specific antibodies were considered abnormal as follows: for anti‐TG threshold—above 115 IU ml^−1^, for anti‐TPO—above 34 IU ml^−1^, for anti‐TSHR—above 1.75 IU L^−1^. Reference values for TSH were 0.27–4.20 μIU ml^−1^.

### 2.3 Statistical analysis

One‐tailed binomial test was used to compare observed diagnoses frequencies with expected values based on epidemiological data. Distributions of symptoms were compared using chi‐square or Fisher's test (depending on the expected counts Sarkhy et al., 2008). Ordinal variables (MGFA scores) were compared using Kruskal–Wallis test. Significance level was set at .05.

## 3 Results

Two hundred and ten patients were diagnosed with EOMG, 112 with LOMG, 21 had thymoma‐MG. A total of 83.8% were seropositive (including all with thymoma‐MG); in 6% of tested patients, anti‐MuSK antibodies were detected.

Demographic characteristics and prevalence of ATD in MG subgroups are presented in Tables 1 and 2. EOMG was significantly more frequent in females (*p* < .001). Prevalence of ATD was similar in seropositive and seronegative MG patients. No ATD was diagnosed in a small group of MuSK‐MG patients. The age at onset (early‐ or late‐onset MG) had no influence on the ratio of patients with different types of ATD. Thymic hyperplasia was diagnosed with similar frequency (45% and 47%) in patients with ATD and in MG without ATD. There was a tendency in the thymoma prevalence across three groups (chi‐square, *p* = .058). Pairwise comparisons showed that significantly higher rates were observed in the group with non‐ATDs as compared with ATD (16% vs. 4%, Fisher's test, *p* = .049) and MG without ATD (16% vs. 5%, Fisher's test, *p* = .049).

**Table 1:** Demographics and clinical characteristics of myasthenia gravis (MG) subgroups

	Early‐onset MG	Late‐onset MG	Thymoma‐MG
Age at disease onset (years)	23.9 (*SD* 11.4)	66.3 (*SD* 9.3)	45.9 (*SD* 11.6)
Mean disease duration (years)	11.4	3.8	4.9
% females	88.1	37.5	42.8

Early‐onset MG was significantly more frequent in females (*p* < .001).

**Table 2:** Characteristics of the studied ATD subgroups and association with different MG groups

	MG without ATD	MG + HT	MG + GD	MG + TAB
*N* (% of total group)	219 (63.8)	31 (9)	15 (4.4)	46 (13.4)
Sex (% of female patients)	63.9	67.7	100	78.1
Age of onset of MG in years (mean, *SD*)	37.8 (22.8)	40.4 (26.1)	42.6 (19.8)	42.0 (21.4)
MG group (*n*) (%)				
Early‐onset MG (210)	63.8	9.0	4.3	13.8
Late‐onset MG (112)	65.2	10.7	4.5	12.5
Thymoma MG (21)	57.1	0	4.8	14.3

F, female; M, male; *SD*, standard deviation; MG, myasthenia gravis; ATD, autoimmune thyroid disease; HT, Hashimoto's thyroiditis; GD, Graves’ disease; TAB, thyroid antibodies.

The clinical characteristics of the studied subgroups and association with different MG subgroups are presented in Table 1.

**Figure 1:**
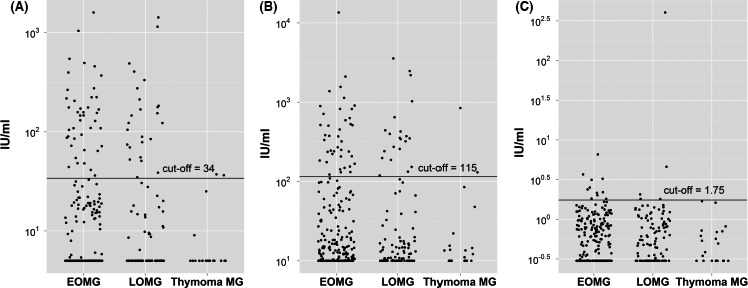
Antithyroid peroxidase antibodies (TPO) (A), antithyroglobulin antibodies (TG) (B), and anti‐TSH receptor autoantibodies (TRAB) (C) results in EOMG, LOMG, and thymoma‐myasthenia gravis. Individual values are given (log scale)

Anti‐TPO, anti‐TG, and TRAB individual values are presented in Figure 1. TRAB were detected in 6% of females and none of the male EOMG, 7.1% females and 2.9% of males with LOMG and none of the thymoma‐MG patients. Comparisons with population reference value (3%) conducted for each subgroup revealed significant difference in EOMG female group only (*p *=* *.024). The prevalence of anti‐TPO and anti‐TG antibodies was compared with values expected for general population (binomial test) (Gardas, 1991). Anti‐TPO antibodies in EOMG were present in 21.1% in females and 12% males, significantly more frequently than predicted (*p* = .005 and .03, respectively). In LOMG, anti‐TPO prevalence was 35.7% in females and 14.3% in males, more frequent than in general population (*p* < .001 for both values). Anti‐TG was present in 20% of females and 12% of males with EOMG (*p* < .001 and *p* = .052, respectively). In LOMG, prevalence of anti‐TG was 26.2% in females and 14.3% in males (*p* < .001). In thymoma‐MG, anti‐TPO and anti‐TG antibodies were present in 11.1% females and 8.3% males (*p = *ns).

### 3.1 Clinical course

At the time of diagnosis, MG was purely ocular in 16.7% of patients with MG alone, 13.3% with MG and ATD, and 9.7% in MG with non‐ATD (*p = *ns).

A total of 80% of patients with GD had ocular symptoms, as compared with 67% with TAB and 58% with HT, but similar to patients with MG only (83%) or MG and non‐ATDs (81%) (pairwise comparison, *p = *ns). Patients had ptosis and/or diplopia attributable to MG. None presented with Graves orbitopathy. When MGFA classification of the severity of MG symptoms in patients with ATD, non‐ATD and patients with no thyroid disease was compared, no significant differences were demonstrated (data not shown).

Myasthenic crisis occurred in 51 patients: 33 (15.9%) with MG only, 6 (21.4%) with non‐ATD, and 12 (13.6%) with ATD. The risk was similar in MG with and without ATD (chi‐square, *p* = .613).

Immunosuppressive therapy (either monotherapy with prednisone or polytherapy) was received by 131 patients with MG only (59.8%), 41 (44.6%) with ATD, and 24 (75%) with non‐ATD. Three groups differed significantly (chi‐square, *p = *.005). Pairwise comparisons showed that immunosuppressants were applied less often in MG + ATD when compared with either of the remaining two groups (MG + ATD vs. MG alone *p* = .014; MG + ATD vs. MG + non‐ATD *p* = .003). Similar pattern emerged when use of more than one immunosuppressant was considered. Significant between‐group differences (chi‐square, *p = *.004) were explained by higher rates of combined immunosuppression in MG without ATD and non‐ATD when compared with MG + ATD group (27.4%, 37.5%, and 12.0%, respectively).

## 4 Discussion

Our study confirms that ATDs in MG patients are more prevalent than in general population (Kanazawa, Shimohata, Tanaka, & Nishizawa, 2007). Reported rate of coexistence of ATD and MG ranged between 5% and 14%, being higher in the studies using laboratory criteria as opposed to data from healthcare registries (Bjoro et al., 2000; Boelaert et al., 2010; Chen, Yeh, & Chiu, 2013; Gardas, 1991; Kanazawa et al., 2007; Nauman, 1991; Szyper‐Kravitz, Marai, & Shoenfeld, 2005). In study by Yeh et al. (2015), allergic conjunctivitis, allergic rhinitis, HT, Graves disease, malignancies, or diabetes mellitus were associated with an increased risk of MG. Thyroid disease was most strongly associated with the subsequent development of MG. In the Japanese study, 11.9% of MG patients had Graves’ disease or Hashimoto's thyroiditis (Kanazawa et al., 2007). In our cohort, 13.4% of the patients had GD or HT. We also demonstrated the presence of antithyroid antibodies in further 13.4% of patients, with similar frequency in early‐ and late‐onset MG. In a recent study, 16% of 114 EOMG, 9% of 44 LOMG, and 17% of 110 thymoma‐MG patients had antibodies against TPO or TG (Klein et al., 2013). Mechanism of such overlap of ATD and MG has not been fully elucidated. Patients with MG or with ATDs and their relatives may express a wide repertoire of organ‐specific antibodies, and other autoimmune diseases are common in MG patients (Keesey, 2004; Klein et al., 2013; Mao et al., 2011). Various HLA haplotypes are associated with EOMG (Toth, McDonald, Oger, & Brownell, 2006; Vandiedonck et al., 2004). HLA‐DR3 antigen may predispose to the development of other autoimmune diseases in EOMG (Toth et al., 2006), and is often seen in Graves’ disease (Simpson, Westerberg, & Magee, 1966; Toth et al., 2006). ATDs significantly increases the risk of ATD in other family members, especially females, while familial MG is rare (Boelaert et al., 2010). Clustering of ATD and MG reflects complex genetic mechanism of autoimmune diseases (Avidan et al., 2014; Cardenas‐Roldan, Rojas‐Villarraga, & Anaya, 2013; Tomer et al., 2015).

Myasthenia gravis patients with ATD in our study less frequently needed immunosuppressive treatment than patients with MG alone, indicating milder course of MG. Similar finding was reported in Italian and Taiwanese study, while such relationship was not observed in the Japanese study (Chen et al., 2013; Kanazawa et al., 2007; Marino et al., 1997). In Nakata et al. (2013) study, seropositive patients with other autoimmune diseases demonstrated less severe MG course.

Coexistence of ATD with ocular MG was reported in several studies (Chen et al., 2013; Marino et al., 1997, 2000). We found significantly more ocular symptoms only in GBS patients, but not in MG in general, as reported in a largest series so far (Chen et al., 2013).

However, we have not found a significant association between ocular myasthenia and thyroid diseases, including ATD. Some authors, especially in Asian MG patients, emphasized higher incidence of ocular MG if ATD coexisted. These results were not confirmed by all authors (Garlepp et al., 1981; Ratanakorn & Vejjajiva, 2002). In the study by Marino et al. (1997), ocular myasthenia was more often observed in patients with ATD. Patients with ocular MG accounted for as much as 40% of those with MG and ATD (Marino et al., 1997). The discrepancies of different studies’ result may be due to several factors including sample size, different diagnostic criteria of ATD, or differences specific to certain populations. Many authors also analyzed the relationship between the MG and thyroid ophthalmopathy. We have not seen Graves’ ophthalmopathy in any of our MG patients. Graves’ ophthalmopathy is reported in approximately 3%–5% of patients with Graves’ disease only (Wiersinga & Bartalena, 2002).

We did not find differences in ATD prevalence between seropositive and seronegative MG (Toth et al., 2006). In Japanese study, other autoimmune disorders occurred more often in seropositive MG patients (24.1%), than in MuSK‐Ab‐positive patients (8.4%) among which ATDs were predominant (Nakata et al., 2013). In previous studies, less frequent seropositivity in MG with ATD could be attributed either to a small number of patients or (Kanazawa et al., 2007) reflected high number (25%) of thymoma patients in the cohort. Thymoma in our study was related to higher risk of non‐ATD (Golden, Robinson, Saldanha, Anton, & Ladenson, 2009; Marino et al., 1997).

Myasthenia gravis severity graded with MGFA scale or occurrence of a myasthenic crisis was similar in our patients with and without ATD, as observed by others (Chen et al., 2013). Similarly, results of treatment in the Kanazawa et al. (2007) study, were not significantly different in patients with and without GBD.

In Meng, Jing, Li, Zhang, and Wang (2016) study, patients with HT and other ATDs were more sensitive to respond to glucocorticoid therapy; there were no significant correlations between the MG remission rate and TSH level, total antibody level, TGAb, and TMAb level.

To conclude: Our study confirms frequent coexistence of thyroid disease in patients with MG and milder course of MG associated with ATD.

## Disclosures

The authors declare no financial or other conflict of interest.

## Conflict of Interest

None declared.

## Funding Information

Polish National Centre of Science (grant/award number: NSC DEC‐2011/01/B/NZ5/05346).
